# Metal-Induced Genotoxic Events: Possible Distinction Between Sporadic and Familial ALS

**DOI:** 10.3390/toxics13060493

**Published:** 2025-06-12

**Authors:** William Wu Kim, Gregory Zarus, Breanna Alman, Patricia Ruiz, Moon Han, Paul Mehta, Chao Ji, Hoormat Qureshi, James Antonini, Mohammad Shoeb

**Affiliations:** 1 Office of Innovation and Analytics (OIA), Agency for Toxic Substances and Disease Registry(ATSDR), Centers for Disease Control and Prevention (CDC), Department of Health and Human Services (HHS), 4770 Buford Highway, Mailstop S106-5, Chamblee, GA 30341, USA; 2 Oak Ridge Institute for Science and Education, Oak Ridge Associated Universities (ORISE), 100 Orau Way, Oak Ridge, TN 37830, USA; 3 Department of Neuroscience, Georgia State University, Atlanta, GA 30303, USA; 4 Health Effects Laboratory Division, National Institute for Occupational Safety and Health, Morgantown, WV 26505, USA

**Keywords:** ALS, biomarkers, metals, telomere, DNA damage, neurotoxicity

## Abstract

Metal exposure is a potential risk factor for amyotrophic lateral sclerosis (ALS). Increasing evidence suggests that elevated levels of DNA damage are present in both familial (fALS) and sporadic (sALS) forms of ALS, characterized by the selective loss of motor neurons in the brain, brainstem, and spinal cord. However, identifying and differentiating initial biomarkers of DNA damage response (DDR) in both forms of ALS remains unclear. The toxicological profiles from the Agency for Toxic Substances and Disease Registry (ATSDR) and our previous studies have demonstrated the influence of metal exposure-induced genotoxicity and neurodegeneration. A comprehensive overview of the ATSDR’s toxicological profiles and the available literature identified 15 metals (aluminum (Al), arsenic (As), cadmium (Cd), chromium (Cr), cobalt (Co), copper (Cu), iron (Fe), lead (Pb), manganese (Mn), mercury (Hg), nickel (Ni), selenium (Se), uranium (U), vanadium (V), and zinc (Zn)) showing exposure-induced genotoxicity indicators associated with ALS pathogenesis. Genetic factors including mutations seen in ALS types and with concomitant metal exposure were distinguished, showing that heavy metal exposure can exacerbate the downstream effect of existing genetic mutations in fALS and may contribute to motor neuron degeneration in sALS. Substantial evidence associates heavy metal exposure to genotoxic endpoints in both forms of ALS; however, a data gap has been observed for several of these endpoints. This review aims to (1) provide a comprehensive overview of metal exposure-induced genotoxicity in ALS patients and experimental models, and its potential role in disease risk, (2) summarize the evidence for DNA damage and associated biomarkers in ALS pathogenesis, (3) discuss possible mechanisms for metal exposure-induced genotoxic contributions to ALS pathogenesis, and (4) explore the potential distinction of genotoxic biomarkers in both forms of ALS. Our findings support the association between metal exposure and ALS, highlighting under or unexplored genotoxic endpoints, signaling key data gaps. Given the high prevalence of sALS and studies showing associations with environmental exposures, understanding the mechanisms and identifying early biomarkers is vital for developing preventative therapies and early interventions. Limitations include variability in exposure assessment and the complexity of gene–environment interactions. Studies focusing on longitudinal exposure assessments, mechanistic studies, and biomarker identification to inform preventative and therapeutic strategies for ALS is warranted.

## 1. Introduction

Amyotrophic lateral sclerosis (ALS) is a progressive and fatal neurodegenerative disorder characterized by the degeneration of motor neurons in the brain, brainstem, and spinal cord [1,2]. This degeneration results in muscle weakness, atrophy, and eventual paralysis, often leading to death within 3–5 years of diagnosis due to respiratory failure.

In the United States, neurological disorders rank second only to cardiac diseases in terms of health burdens associated with environmental exposures [3], and ALS alone affects approximately 30,000 individuals at a prevalence rate of 9.1 per 100,000 population, with 1.5–1.7 new cases per 100,000 each year [4,5]. The prevalence of ALS is expected to increase as the population ages [6]. Despite low frequency in case diagnosis, ALS shares many pathological similarities with other neurodegenerative diseases, such as primary lateral sclerosis, multiple sclerosis, and Parkinson’s disease, making its study relevant across multiple fields of neurodegenerative research [7,8].

ALS can be largely classified as familial (fALS) or sporadic ALS (sALS). fALS accounts for roughly 5–10% of all cases due to inheritance of specific gene mutation(s), while the remaining 90–95% are classified as sALS. Although sALS lacks a clear familial inheritance pattern, recent research has identified multiple novel mutations in protein-coding genes among sALS patients, suggesting a polygenic basis that may interact with environmental exposures [9,10,11,12]. Individuals with higher mutational loads in these genes show significantly increased probabilities of developing ALS [12]. Despite the categorization, there is considerable overlap between these forms, as pathogenic mutations observed in fALS, such as those in superoxide dismutase 1 (SOD1), TAR DNA-binding protein (TARDBP), fused in sarcoma (FUS), and chromosome 9 open reading frame 72 (C9orf72), are frequently identified in sporadic cases as well [10,11]. Since 1993, more than 40 ALS-related genes have been discovered, providing crucial insights into the disease’s pathology. Collectively, mutations in SOD1, TARDBP, FUS, and C9orf72 account for approximately 70% of fALS and 15% of sALS cases, effectively explaining one in six ALS cases [10,13].

In 2008, the United States (US) Congress passed Public Law 110–373 and tasked the Centers for Disease Control and Prevention and the Agency for Toxic Substances and Disease Registry (CDC/ATSDR) to launch a national population-based registry. Since then, the National ALS Registry has evolved into a multifaceted research platform which includes the National ALS Biorepository to analyze genetics, determine disease progression, and identify biomarkers. Researchers can request samples from the Biorepository to augment their analyses. The objectives of the Registry are multifold and include examining the epidemiology of ALS (e.g., incidence, prevalence, and mortality), determining patient demographics, and examining and identifying possible etiologies.

While genetic mutations and deregulation are central to ALS development, environmental metal exposures are increasingly implicated in the etiology of sALS. Metals such as aluminum (Al), arsenic (As), cadmium (Cd), chromium (Cr), cobalt (Co), copper (Cu), iron (Fe), lead (Pb), manganese (Mn), mercury (Hg), nickel (Ni), selenium (Se), uranium (U), vanadium (V), and zinc (Zn) are widely hypothesized to contribute to ALS pathogenesis. These metals are commonly found in hazardous waste sites and occupational settings, with all except Fe listed on the Agency for Toxic Substances and Disease Registry (ATSDR)’s Substance Priority List (SPL) [14]. Metals pose significant environmental and occupational hazards due to their ability to persist in the environment and bioaccumulate in the brain and spinal cord, key sites of ALS neuropathology. Despite growing evidence of an association between metal exposure and ALS [7,15,16], the precise mechanisms and the extent to which such exposures may contribute to disease progression remains unclear. Mechanistically, metal exposure may contribute to ALS through several interrelated pathways. Redox-active metals like copper and iron can elevate oxidative stress, damaging DNA and proteins [17,18]. Disruption of mitochondrial function impairs energy metabolism and promotes apoptosis [19]. Metals also influence protein misfolding, especially of SOD1 and TDP-43 [7,20], and can interfere with DNA repair systems, compounding genomic instability in neurons [21].

The ATSDR has developed detailed toxicological profiles for each of these metals, summarizing their genotoxic potential and health risks [22]. These profiles provide critical insight on metals-induced DNA damage and other genotoxic endpoints. However, while the ATSDR’s profiles often mention the role of metals in neurodegenerative diseases like ALS, they lack significant details in the literature on how the genotoxic properties of these metals may drive the disease pathogenesis. For instance, while metals are known to induce DNA damage, oxidative stress, and epigenetic alterations [14], the precise mechanisms by which they contribute to motor neuron degeneration in ALS remain poorly understood. This gap in the literature hinders efforts to establish a direct causal association between metal exposure and ALS progression, and it underscores the need for a more thorough exploration of metal’s role in genotoxicity within the context of ALS.

Current research suggests metals contribute to genotoxicity in ALS [23], leading to DNA damage and subsequent motor neuron degeneration [19,21,23]. Genotoxic endpoints refer to measurable alterations in DNA integrity, including DNA strand breaks, point mutations, chromosomal abnormalities, and epigenetic modifications such as DNA methylation [24]. These effects are relevant to ALS, where the loss and eventual death of motor neurons is closely tied to cellular dysfunction. However, “DNA damage” is a broad term, encompassing various forms of genomic instability. Identifying specific genotoxic endpoints and biomarkers of DNA damage response (DDR) associated with ALS is essential to understand how metals can disrupt biological processes and contribute to ALS. The ATSDR has identified 11 unique genotoxic endpoints that collectively define “DNA damage” [24]. These endpoints will serve as the framework for categorizing genotoxic events related to metal’s possible involvement in ALS.

Research associating metal-exposure with ALS is complicated due to variety of metals involved and the varying degrees of toxicity they exhibit. Each metal’s impact on ALS likely differs based on factors such as ion species, oxidation state, and duration of exposure [18,20,25,26]. Despite this complexity, most studies to date have focused on individual metals or small groups of metals, often failing to provide a comprehensive overview of the broader effects of metal exposure on ALS. This lack of systematic analysis has hindered the development of conclusive mechanisms for how these metals may cause ALS. Moreover, distinguishing between the roles of metals in fALS versus sALS remains challenging, as metal exposure is primarily environmental, often occupational, and thus not directly inherited [15,27,28,29]. However, metals may exacerbate the downstream effect of existing genetic mutations in fALS cases or contribute to motor neuron degeneration or heterogeneity in symptoms presented in sALS cases. For instance, metal-induced oxidative stress and disruption of metal homeostasis could intensify the pathogenic effects of mutations in ALS-associated genes such as SOD1 and TDP-43 [17,25]. Recent evidence from a forthcoming National ALS Registry study further supports the multi-step model of ALS pathogenesis, highlighting the cumulative interplay between environmental exposures and genetic susceptibilities, offering critical insights into the progression and potential intervention points for ALS [30]. Therefore, it is crucial to elevate the understanding of how metals may contribute to both forms of ALS and to identify unique genotoxic biomarkers for each form.

This review aims to provide a comprehensive analysis of the role of metal exposure in ALS pathogenesis. Specifically, it seeks to (1) provide evidence suggesting metal exposure in fALS and sALS; (2) summarize the genotoxic endpoints and biomarkers of DDR associated with ALS; (3) surmise potential mechanisms of metal-induced genotoxicity contributing to motor neuron degeneration; and (4) potential distinctions in genotoxic biomarkers between fALS and sALS. This analysis highlights critical data gaps and underscores the importance of identifying early biomarkers of DDR to better understand the environmental and genetic interplay in ALS, paving the way for preventative strategies and therapeutic interventions.

## 2. Methodology

### 2.1. Search Strategy

This review investigates the possible contributions of heavy metal exposure on the pathogenesis of ALS, focusing on those genotoxic events both have in common and consideration of potential genetic contributions from metal exposure specifically. This study was conducted by synthesizing the literature on ATSDR’s toxicological profiles alongside a comprehensive database literature search, enabling a thorough examination of the potential genotoxic endpoints associated with various metal exposures and finding commonality in those associated with ALS.

The primary sources for this review include toxicological profiles published by the U.S. Agency for Toxic Substances and Disease Registry (ATSDR) [31], which summarize health effects of hazardous substances. These federal documents were initially examined for information regarding the relationship between specific metals and ALS. As outlined by Zarus et al (2024), ATSDR systematically compiles and reviews all published research on the health effects of various substances, making their toxicological profiles a reliable starting point. Nonetheless, peer-reviewed literature beyond these profiles served as the primary data source for this review [32].

A comprehensive literature search was conducted using databases including PubMed, Scopus, Embase, Medline, Google Scholar, and the Environmental Science Collection to supplement the limited ALS-related data in the toxicological profiles. The search, performed through May 2024, focused on studies examining the relationship between ALS and exposure to 15 metals: Al, As, Cd, Cr, Co, Cu, Fe, Pb, Mn, Hg, Ni, Se, U, V, and Zn. All metals, except Fe, were selected based on their inclusion in the ATSDR’s Substance Priority List (SPL), which ranks hazardous substances commonly found at National Priorities List (NPL) sites based on their toxicity, frequency of occurrence, and potential for human exposure [14].

The SPL is periodically updated to reflect new evidence, with the ATSDR systematically preparing toxicological profiles for each substance listed. Each of the 14 SPL-listed metals, excluding Fe, has a toxicological profile developed by the ATSDR due to their significant toxic potential. Literature searches were restricted to studies published in English and combined the metal names with keywords such as “[Metal Name] Amyotrophic lateral sclerosis” and “[Metal Name] ALS”. The detailed search strategy is provided in Supplementary Table S1.

### 2.2. Study Selection

Studies included in this review provided evidence of significant DNA damage, altered metal homeostasis, or neurodegenerative effects associated with metal exposure in ALS models or affected patients. To ensure clarity and avoid overlap, studies were categorized into four groups: In vitro, In vivo, Epidemiological, and Hybrid. The Hybrid category represents studies that combined both in vitro and in vivo experiments, providing a comprehensive perspective by integrating mechanistic insights from both approaches. The Epidemiological category encompasses traditionally classified epidemiological studies as well as those with a primary epidemiological focus that include supplementary in vitro or in vivo components, enabling validation of epidemiological findings and reducing data heterogeneity. All in vitro and in vivo studies were included regardless of the population type to capture the breadth of mechanistic insights across experimental systems.

This review focuses on 15 unique metals; however, individual studies included in the analysis may examine only one or a subset of these metals. To ensure clarity and precision, only studies that analyzed the possible contributions of DNA damage induced by individual metal exposures, particularly damage consistent with that observed in ALS pathogenesis, were included. Studies that assessed the combined effects of multiple metals without isolating individual contributions were excluded. Similarly, research on heavy metal exposure in other neurodegenerative diseases, such as Frontotemporal Disease (FTD), Gulf War illness, Alzheimer’s disease, or Parkinson’s disease, was excluded to ensure specificity to ALS. Additionally, review articles were excluded to ensure the inclusion of primary data sources and maintain the rigor of the analysis.

This approach was designed to enhance the validity and precision of the review’s findings by isolating the genotoxic and neurodegenerative properties of individual metals in common with ALS, with the selection criteria summarized in a PECOS (Population, Exposure, Comparator, Outcome, and Study) framework in Supplementary Table S2.

### 2.3. Data Extraction

For each study included in this review, data were extracted on author, title, study design, metal type/ion species, exposure level, duration, and results. Key findings included evidence for DNA damage, biomarkers, mutations in ALS-related genes, and the observed effects or relationships between each metal and ALS models or patients. The term “DNA damage” was further categorized into specific genotoxic endpoints based on Shoeb et al. (2023) [24], providing a detailed framework for assessing DNA integrity. Biomarkers were defined as measurable indicators of processes such as genotoxic endpoints, neurodegenerative effects, or altered metal homeostasis, and were identified through a team-based consensus approach following a systematic review of all included studies. Mutations in ALS-related genes were determined using Wang et al. (2023) [13], which catalogs all known ALS genes as of 2023.

Significant associations between metal exposure and genotoxicity were identified in cases where studies explicitly reported a *p*-value < 0.05 or when a confidence interval (95% CI or higher) excluded the null value. If no statistical test was performed, the determination of significance relied on the author’s interpretation, which was informed by alternative experimental approaches broadly categorized into imaging and visualization techniques, protein and molecular characterization methods, analytical and quantitative assays, and clinical or observational assessments. Non statistically significant associations were recorded when studies explicitly stated no significant relationship or when an association was tested but not described as significant, indicated by a *p*-value > 0.05 or a confidence interval (<95% CI) including the null value.

This approach enabled a systematic assessment of the evidence, highlighting the individual contributions of each metal to ALS pathogenesis and uncovering patterns and discrepancies in the literature. Data were organized into structured tables and visualized using tools from the Office Suite, ensuring clarity and accessibility for the findings presented.

## 3. Results

### 3.1. Toxicological Profile Findings

Among the 14 metals with ATSDR toxicological profiles, only Al, Pb, Mn, and Se briefly mention ALS, each in a short paragraph with limited detail [31]. Al was associated with ALS clusters in Guam and similar regions, though findings are confounded by co-exposures and calcium (Ca) deficiency, limiting interpretation. Pb was associated with increased ALS risk at low blood lead levels, but this is based on general neurotoxicity data, not ALS-specific studies. Mn was elevated in ALS spinal cord samples, but use of a small sample size and reverse causality may have affected the results. Se was tied to higher ALS incidence in a Se-exposed population, though low exposure levels, lack of individual data, and confounders limit conclusions. No ALS mention appears for As, Cd, Cr, Co, Cu, Hg, Ni, U, V, or Zn, and no ATSDR profile exists for Fe.

### 3.2. Summary of Studies

**Figure 1:**
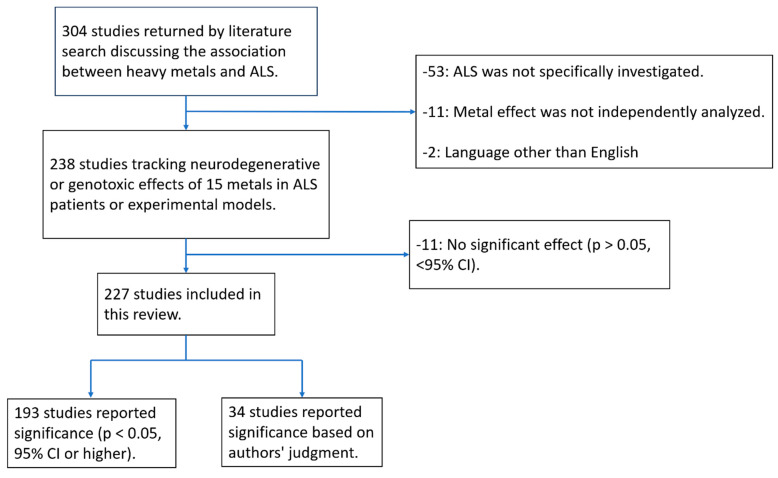
Study Selection Flowchart displays the study screening pipeline.

A total of 304 studies investigating the relationship between exposure to 15 metals and outcomes in ALS patients or ALS models were initially identified. After excluding studies that did not specifically investigate ALS (53 studies), did not independently analyze metal effects (11 studies), or were written in languages other than English (2 studies), 238 studies remained for review. Of these, 11 studies reported no significant association between metals and ALS, leaving 227 studies that met the criteria for inclusion (Figure 1).

Table 1 summarizes these 227 studies, highlighting the individual metals investigated, their Substance Priority List (SPL) ranks (where available), and study types. Next, a vote-counting approach was applied to assess the directional relationships between heavy metal exposure and ALS. Each study was assigned a score of +1 for reporting a significant positive or negative association with ALS, based on evidence of DNA damage, altered metal homeostasis, or neurodegenerative effects. For studies analyzing multiple metals, each metal with a significant association was scored independently. Among the studies, 94 were experimental (31 In vitro, 52 In vivo, and 11 Hybrid studies), while 133 were epidemiological.

Pb and Cu (34 studies each) and Zn (31 studies) were the most frequently associated with significant findings, followed by Fe (24), Se (20), and Mn (17). Hg (18 studies), Cd (15), and Al (13) showed intermediate levels of association, while Ni (8), Co (5), As (3), Cr (2), V (2), and U (1) were associated with fewer studies.

Notably, Cu, Fe, Ni, Se, V, and Zn were associated with potential therapeutic effects for ALS, as indicated by an asterisk (*). The total number of included studies (227) is emphasized in the bright yellow cell at the bottom of Table 1.

### 3.3. Metal-Induced Genotoxicity in ALS

Among the 15 metals analyzed, Cd was the only one directly observed to induce DNA damage in studies involving ALS patients or ALS model systems, with genotoxic endpoints including DNA methylation, inhibition of DNA repair processes, and inferred DNA fragmentation. DNA damage, specifically DNA fragmentation, was inferred for Co, Cu, Fe, Se, and Zn, based on evidence of processes that occur upstream of DNA fragmentation, such as oxidative stress, reactive oxygen species production, and neural cell apoptosis (Supplementary Table S4). DNA fragmentation for cadmium was similarly inferred based on these upstream processes (Supplementary Table S4). These findings are summarized in Table 2, which categorizes metals into “Yes” (direct evidence of DNA damage), “Inferred” (suggested by potential biomarkers or test outcomes), and “Not Specified” (no observed evidence). Specific genotoxic endpoints, such as DNA methylation and inferred DNA fragmentation, are listed for each metal in the “Evidence” column, with cell highlights—green (Yes), yellow (Inferred), and red (Not Specified)—indicating the level of evidence.

DNA damage was not directly observed in studies conducted in confirmed ALS patients or ALS-specific experimental models for Al, As, Cr, Pb, Mn, Hg, Ni, U, or V, as none of the 11 genotoxic endpoints that collectively define “DNA damage” were reported in these ALS-focused studies. Supplementary Table S3 provides a detailed framework for these endpoints, which include chromosomal damage, DNA methylation, DNA–protein cross-linking, micronuclei formation, sister chromatid exchange, DNA repair inhibition, gene mutation, chromosomal aberration, DNA fragmentation, DNA strand breaks, and telomere alteration. Among these, DNA methylation, DNA repair inhibition, and inferred DNA fragmentation were the only genotoxic endpoints observed following exposure to the 15 metals, while the remaining eight endpoints were not reported in studies involving metal exposure in confirmed ALS patients or ALS-specific experimental models.

To further support these findings, Supplementary Table S4 summarizes test outcomes and potential biomarkers for each metal. These outcomes were used to match biomarkers to the genotoxic endpoints listed in Table 2, strengthening the evidence for direct or inferred DNA damage following metal exposure.

### 3.4. Metal Associations with ALS Mutations

Five ALS-related genes—C9orf72, SOD1, TDP43, TBK1, and TUBA4A—were identified in studies involving confirmed ALS patients or ALS-specific experimental models with documented heavy metal exposure. These genes exhibited mutations or disruptions consistent with those reported in broader ALS genetic studies not specifically focused on environmental exposures [13]. Table 3 summarizes these findings, listing the mutations associated with each metal and gene alongside their effects in ALS models or patients. SOD1 exhibited the highest number of unique mutations, with 23 variants linked to neurodegenerative effects or altered metal homeostasis following metal exposure. The most frequently studied mutation, SOD1(G93A), appeared in 28 studies, followed by SOD1(G37R), SOD1(G85R), SOD1(H46R), and SOD1(AV4), with 10, 8, 8, and 6 studies, respectively. In contrast, TUBA4A was linked to two unique mutations (A383T and R320C), while C9orf72, TDP43, and TBK1 were each associated with a single mutation. Importantly, while 9 of the 15 metals were matched to neurodegenerative effects or altered metal homeostasis in ALS models or patients with these mutations, none of the metals were reported to cause the mutations themselves.

Cu and Zn were the most frequently studied metals, with 34 and 30 studies, respectively, matching these metals to neurodegeneration or altered metal homeostasis in ALS models or patients with known ALS mutations. Table 3 highlights that these findings primarily focused on SOD1 mutations or metal substitutions, with the exception of TDP43(A314T), which was observed in association with both Cu and Zn. Co and Fe followed with 13 and 9 studies, respectively, all linked to SOD1 mutations such as G85R, G93A, H46R, and G93E. Fe’s most frequently studied mutation was SOD1(G93A), while Co’s effects were distributed across several mutations.

Se (3 studies), Ni (2), Hg (1), Mn (1), and Pb (1) were linked to fewer studies involving specific ALS mutations. Se was uniquely associated with mutations in TUBA4A (A383T and R320C), while Hg was linked to a deletion mutation in TBK1 (c.1852_1854delGAA: p.E618del). Pb was associated with TDP43(A314T), consistent with findings for Cu and Zn. No mutations were observed in association with Al, As, Cd, Cr, U, or V in the studies reviewed.

Table 3 uses color-coded cells to indicate the frequency of studies matching each metal to ALS mutations: white (0 studies), green (1 study), orange (2–9 studies), and red (>9 studies). Metals without any observed mutations, such as Al, As, Cd, Cr, U, and V, are not included. The total number of studies reviewed for each mutation and metal is summarized, with the bottom-right yellow cell showing the total studies included (95). Supplementary Table S5 complements Table 3 by providing detailed information on all mutations associated with each metal in ALS patients or model systems, including their respective citations, to further support and contextualize the findings.

## 4. Discussion

### 4.1. Metal Exposure-Induced Genotoxicity in ALS; Unexplored Genotoxic Endpoints

The aging of the global population is a primary risk factor for a variety of neurodegenerative diseases, including ALS, sometimes referred to as motor neuron disease [6]. Genetics and environmental exposures, including metals, may influence ALS pathogenesis. The role of DNA damage in ALS pathogenesis has long been recognized, yet the specific genotoxic mechanisms that contribute to motor neuron degeneration remain poorly understood. While studies have demonstrated increased genomic instability in ALS patients, “DNA damage” is broadly defined and encompasses a range of molecular events. Prior to this study, no research had systematically categorized the specific genotoxic endpoints relevant to ALS. By applying the 11 genotoxic endpoints outlined by the ATSDR, this study provides the first structured analysis of how heavy metal exposure contributes to ALS-related genomic instability. Genotoxic endpoints and their associated biomarkers have been the focus of ALS research for decades; however, no definitive data currently distinguish between familial ALS (fALS) and sporadic ALS (sALS) following exposure to any specific metal. Some potential differences between fALS and sALS in response to metal exposures are summarized in Table 4.

Epidemiological association of metals, such as Hg, Pb, and Se, have been suggested in ALS [33], significantly elevated concentrations of other metals, such as Al, Cd, Co, Cu, Mn, Pb, U, V and Zn, have also been reported [34]. All 15 metals discussed in this study were previously noted in the ATSDR’s toxicological profiles and/or related literature as capable of independently inducing genotoxic effects, further reinforcing the need to assess their role in ALS. Understanding and identifying metal-induced genotoxic biomarkers in the prodromal or progression phases of ALS may help in targeted therapeutic strategies aimed at mitigating symptoms and neurodegeneration due to genomic instability.

Metal exposure, whether through the occupational or non-occupational environment, increases the risk for ALS [35]. There may be a time delay between exposure and onset of ALS symptoms, which may initiate pathogenic processes leading to the development of clinical phenotype. That is where the DNA damage caused by hazardous exposures such as metals comes in, failure to repair damage and inability to regenerate cells may be due to (1) dysfunction of DNA polymerase enzymes resulting in elongated 3′ overhang and cellular apoptosis (2) dysfunctional or bypass of DNA repair and (3) inability to regenerate new cells because of the post-mitotic nature of these cells [36]. Despite the huge time lag (therapeutic window), the major hurdle for researchers is to identify primary genotoxic biomarkers specifically for sALS and to prevent exposure at first place.

The presence of metals such as Cu, Se, and Zn have been reported in plasma and urine samples of ALS patients [37]. Accumulation of Hg was identified in degenerating motor neurons of ALS patients through post-mortem autopsies, and Pb, Hg and Mg were speculated to be involved in ALS onset [34]. Of the 11 established genotoxic endpoints [24], only DNA methylation, DNA repair inhibition, and inferred DNA fragmentation have been reported in studies of metal exposure in ALS patients or ALS-specific experimental models (Table 2). The lack of research identifying chromosomal aberrations, DNA strand breaks, sister chromatid exchange, telomere shortening, and other key genotoxic endpoints suggests that the role of metal-induced genotoxicity in ALS has yet to be fully characterized; all 15 metals examined in this study have been suggested as genotoxic in toxicological profiles and related literature; however, several endpoints remain unidentified (Supplementary Tables S1–S3). This data gap highlights the need for future research to systematically assess these overlooked endpoints using experimental models, patient-derived tissues, and advanced genomic tools to determine their relevance in ALS pathology.

### 4.2. Genotoxic Biomarkers and Early Diagnosis Potential

Beyond the identification of underexplored genotoxic mechanisms, this study also establishes a foundation for future biomarker discovery. If specific genotoxic endpoints can be linked to ALS progression, early biomarkers may be identified based on measurable molecular signatures of DNA damage response (DDR). For instance, DNA strand breaks could be detected through increased γH2AX and 53BP1 phosphorylation, while chromosomal aberrations might be observed through elevated micronucleus formation in patient-derived cells. Establishing clear mechanistic associations between specific test outcomes and defined genotoxic endpoints would represent a major step forward in the development of ALS biomarkers, potentially allowing for earlier and targeted therapeutic interventions aimed at reducing genomic instability.

### 4.3. Metal Exposure and ALS-Linked Genetic Mutations

In addition to genotoxicity, another critical finding of this study is the association between metal exposure and ALS-linked mutations. This study is among the first to identify distinct patterns in how metals interact with ALS-related genes, revealing that certain metals have strong associations with genetic mutations while others do not. SOD1 mutations were found to be the most frequently associated with metal exposure, with 23 unique variants linked to toxic interactions with cobalt, copper, and zinc (Table 3). Furthermore, pathological similarities, for instance distribution of SOD1 gene, identified as the first ALS-associated gene were observed in both forms of ALS [38,39]. Research suggested that wild-type SOD1 protects against cell damage, by changing its physiological conformation compared to mutant SOD1 in sALS pathogenesis [40,41,42]. This finding reinforces the hypothesis that metal-induced oxidative stress may exacerbate SOD1 misfolding and aggregation, a well-established hallmark of ALS pathology. Similarly, mutations in TDP43, TBK1, and C9orf72 were found to interact with specific metals, including Hg, Pb, Ni, and Se, suggesting that environmental exposure may selectively influence ALS pathogenesis in genetically susceptible individuals (Table 3).

### 4.4. Divergent Associations of Key Metals in ALS

While most metals demonstrated either a positive or negative association with ALS, Zn, Cu, Se, and Fe exhibited both protective and detrimental, contrasting effects, indicating a context-dependent role in disease progression.

Zn is an essential cofactor for Cu, Zn SOD1 and supports antioxidant defense and neuronal stability. Lower Zn levels in blood have been associated with increased ALS risk, particularly in patients with reduced functional status [15,43]. In contrast, higher Zn levels have been reported in cerebrospinal fluid (CSF), spinal cord, hair, and teeth of ALS patients and models, and have been linked to greater disease severity and cellular toxicity depending on tissue, timing, and chemical form [25,44,45,46]. These findings suggest that Zn dysregulation, whether due to deficiency or accumulation, may contribute to both ALS susceptibility and variability in disease progression.

Cu, a critical cofactor for antioxidant enzymes, also shows context-dependent effects. Cu levels are often decreased in ALS CSF, especially in spinal-onset cases, indicating localized deficiency [47,48,49]. However, elevated Cu has been reported in blood, spinal cord, and muscle tissue [50,51,52,53,54], and enriched ⁶⁵Cu isotopic signatures suggest altered Cu metabolism [55]. In SOD1 transgenic models, Cu accumulation and dyshomeostasis correlate with disease progression [53,54,56,57]. Therapeutically, Cu supplementation with copper(II) diacetyl-bis(N^4^-methylthiosemicarbazonato) (CuATSM) extended survival [58,59,60], highlighting that both deficiency and excess of Cu may contribute to neurodegeneration.

Se has been inversely associated with ALS risk in several epidemiological studies, particularly when measured in blood or air samples, suggesting a potential protective role [43,61]. However, lower Se levels have also been linked to increased disease severity in ALS patients, raising the question of whether Se depletion contributes to disease progression or reflects a consequence of advancing pathology [62,63]. Additionally, elevated concentrations of specific Se species such as selenite (SeO_3_^2−^) and selenomethionine (SeMet) have been observed in the CSF of ALS patients, particularly in those with disease-associated genetic mutations, suggesting that Se dysregulation through toxic species may also play a role in ALS pathogenesis [64,65].

Fe, essential for oxygen transport and mitochondrial function, is dysregulated in ALS-affected tissues [66,67]. Elevated Fe levels have been observed in the motor cortex, spinal cord, CSF, and peripheral tissues of ALS patients and models, contributing to oxidative stress, microglial activation, and motor neuron degeneration [68,69,70]. Increased serum ferritin and inappropriate Fe ligands in CSF further support a role for iron mismanagement in disease progression [71,72]. Despite this, Fe-based therapeutics such as iron(III) meso-tetra(4-carboxyphenyl)porphyrin chloride (FeTCPP) have shown neuroprotective effects by scavenging reactive oxygen and nitrogen species and extending survival in SOD1 ALS models [73], suggesting that the impact of Fe depends on its biochemical form and compartmentalization.

These findings suggest that while these metals play essential roles in neuronal homeostasis, their dysregulation may contribute to disease progression, highlighting the need for therapeutic strategies aimed at restoring metal balance.

### 4.5. A Potential Framework for Distinguishing fALS and sALS

One of the most striking implications of these findings is the potential to distinguish between fALS and sALS based on metal exposure patterns. The inability to clearly delineate the contributions of genetic and environmental factors in ALS pathogenesis has long been a challenge in ALS research. While fALS and sALS are typically classified as distinct entities based on inheritance patterns, emerging evidence suggests considerable overlap, particularly in regard to shared molecular mechanisms and genetic variants. The specific role of metal exposure in influencing disease onset and progression within each form remains poorly understood. This study presents a novel hypothesis: metals that exhibit strong interactions with genetic mutations observed in ALS patients or ALS-specific models may accelerate disease onset in genetically predisposed individuals, making them more relevant to familial ALS (fALS). In contrast, metals that show no association with ALS-related mutations may act as independent risk factors, more relevant to sALS. For example, Cu, Zn, and Pb, which have well-documented interactions with ALS-associated genes, may be key contributors to fALS progression, whereas As, Al, Cr, U, and V, which show no clear genetic interactions, may primarily drive sALS. Future studies investigating this distinction could lead to a revised framework for ALS classification, allowing researchers and clinicians to assess environmental risk factors in the context of genetic predisposition, ultimately paving the way for more personalized approaches to ALS diagnosis and treatment.

## 5. Studies Reporting No Statistically Significant Association

While this study identifies significant associations between metal exposure and ALS, 11 studies in the literature did not meet the statistical threshold of *p* < 0.05. However, many reported marginal significance (0.05 < *p* < 0.10) or trends toward increased metal levels in ALS patients. These findings likely contributed to sample size limitations, biological variability, and methodological differences. Several studies observed slightly elevated Co, Fe, Al, Se, and Zn in ALS-affected tissues, but the results did not reach statistical significance due to small sample sizes or measurement inconsistencies. Variability in metal concentrations across biological matrices further complicates exposure assessment, as ALS-related metal dysregulation may be tissue-specific. Some studies detected changes in metal distribution rather than absolute concentrations, suggesting that altered metal localization may play a role in ALS pathology even when overall levels do not appear significantly different.

The lack of statistical significance in these studies does not contradict the broader hypothesis that metal exposure contributes to ALS pathogenesis but underscores the complexity of metal interactions in ALS and the need for standardized exposure assessments.

### Challenges and Future Directions

While these findings offer new insights into ALS pathogenesis, several challenges remain. The variability in exposure assessments across epidemiological studies presents a significant limitation, as different methodologies, including self-reported data, indirect environmental measurements, and direct biomonitoring studies, have been used to assess metal exposure in ALS patients. While longitudinal studies incorporating metal biomarkers in blood, cerebrospinal fluid, and postmortem spinal cord tissue have provided critical insights into metal accumulation in ALS, differences in study design and exposure metrics make establishing a standardized threshold for neurotoxic effects challenging. Further research is needed to refine exposure assessment techniques, ensuring that environmental and biomonitoring data are integrated into ALS risk models with greater precision. Additionally, further studies are required to clarify whether metal exposure is a primary initiator of ALS pathology or merely an exacerbating factor in already predisposed patients. Advanced computational toxicology tools, such as the Comparative Toxicogenomics Database (CTD), may provide valuable insights by identifying novel gene-environment interactions and predicting previously unrecognized molecular pathways influenced by metals.

Future research should aim to validate metal-specific genotoxic signatures in ALS, including biomarkers such as micronucleus formation and DNA double-strand break markers, using patient-derived cells or in vivo models [74]. Investigating metal-modulating therapies, including chelating agents and compounds like CuATSM, may offer targeted intervention opportunities, particularly for genetically stratified ALS subgroups [75]. Emerging strategies such as ferroptosis inhibition—exemplified by compounds like liproxstatin-1—and neuroprotective agents like lithium warrant further evaluation [76,77]. The toxicity profiles of rare earth elements, including lanthanum, also remain understudied [78]. Moreover, metal-induced epigenetic modifications, especially DNA methylation changes, represent a critical frontier in ALS research [79]. Finally, public health efforts to reduce occupational and environmental metal exposure should be prioritized, particularly in populations with known or suspected genetic vulnerabilities [37].

## 6. Conclusions

This review presents a significant advancement in understanding heavy metal exposure as a potential driver of ALS pathogenesis, discussing gene–environment interactions and genotoxic events. By systematically categorizing metal exposure-induced indicators of genotoxicity using the ATSDR’s genotoxic framework, this review identifies a major data gap in ALS research, revealing that nearly 80% of known genotoxic endpoints remain unexplored in ALS. The findings strongly suggest that DNA damage in ALS is not a singular event but rather a complex interplay of multiple genotoxic and environmental mechanisms, many of which are not yet investigated (Table 2, Table 3 and Table 4). The identification of these specific genotoxic endpoints provides a crucial foundation for future biomarker discovery, which could facilitate earlier diagnosis, risk assessment, and therapeutic intervention in ALS.

Additionally, this review reveals clear patterns in the association between metal exposure and ALS-linked genetic mutations, suggesting that gene–environment interactions may influence disease onset and progression. While this observation highlights potentially unique features of ALS, it is important to note that similar disruptions in metal homeostasis, particularly involving Cu, Fe, and Zn, are also seen in other neurodegenerative diseases such as Alzheimer’s and Parkinson’s disease [80,81,82]. These metals are known to promote oxidative stress and protein misfolding in multiple neurodegenerative disorders [83,84], suggesting that the gene–metal interactions described in ALS may reflect shared pathogenic mechanisms rather than disease-specific pathways. The observation that certain metals (Co, Cu, Zn) show strong associations with genetic mutations in ALS patients or ALS-specific model studies, while others (As, Al, U) do not, raises the possibility that metal exposure could serve as a differentiating factor between fALS and sALS (Table 3). This distinction may be further influenced by alterations in metal homeostasis, a phenomenon that also plays a central role in other diseases such as Alzheimer’s and Parkinson’s [80,81]. Future research should examine whether these metal-specific patterns in ALS represent distinct disease subtypes or reflect broader neurodegenerative mechanisms involving gene–environment interactions.

Despite these advancements, critical knowledge gaps remain. Future research should focus on longitudinal biomonitoring studies to quantify metal exposure in ALS patients, mechanistic studies on unexplored genotoxic endpoints, and computational modeling to identify additional gene-metal interactions. The potential to develop early diagnostic biomarkers based on metal-induced DNA damage represents a particularly exciting avenue for future research. Given the growing body of evidence linking environmental exposures to neurodegenerative diseases, public health initiatives should emphasize the need for reducing occupational and environmental exposure to neurotoxic metals, particularly in populations with known genetic risk factors for ALS.

By addressing longstanding gaps in ALS genotoxicity research, this review provides a framework for future investigations, paving the way for early detection strategies, targeted therapies, and a deeper understanding of how environmental factors shape neurodegenerative disease progression.

## Figures and Tables

**Table 1:** Studies on Metal Exposure in ALS.

Metals	SPL Rank	In Vitro	In Vivo	Epidemiological	Hybrid	Total
Arsenic (As)	1			3		3
Lead (Pb)	2			32	2	34
Mercury (Hg)	3		8	9	1	18
Cadmium (Cd)	7	7		8		15
Cobalt (Co)	51	3		1	1	5
Nickel (Ni) *	57	1	1	5	1	8
Zinc (Zn) *	74	8	9	12	2	31
Chromium (Cr)	78			2		2
Uranium (U)	99		1			1
Copper (Cu) *	120	4	14	15	1	34
Manganese (Mn)	143		2	15		17
Selenium (Se) *	151	2	1	16	1	20
Aluminum (Al)	188	3	6	4		13
Vanadium (V) *	208	1	1			2
Iron (Fe) *		2	9	11	2	24
Total		31	52	133	11	227

Note. Table 1 displays studies meeting inclusion criteria, organized by study type (In vitro, In vivo, Epidemiological, Hybrid) to assess consistency across systems and populations. For the Substance Priority List (SPL) rank: 1 represents the highest priority. Iron (Fe) lacks an SPL rank. Metals with potential therapeutic effects for ALS are marked with ‘*’. Totals for each metal and study type are summarized. Cell highlights: green (1–2 studies), orange (3–16 studies), red (>16 studies), excluding “Total” cells. A lack of shading indicates no studies were found. The bottom-right cell in bright yellow shows the total studies included (227). All included studies were performed in ALS patients or ALS model systems, and inclusion criteria are described in the Methodology section.

**Table 2:** Genotoxic Evidence of Heavy Metal Exposure in ALS.

Metal	Genotoxic Endpoints	Evidence
Aluminum (Al)	Not Specified	
Arsenic (Ar)	Not Specified	
Cadmium (Cd)	Yes	DNA Methylation, DNA Repair Inhibition, DNA Fragmentation
Chromium (Cr)	Not Specified	
Cobalt (Co)	Inferred	DNA Fragmentation
Copper (Cu) *	Inferred	DNA Fragmentation
Iron (Fe) *	Inferred	DNA Fragmentation
Lead (Pb)	Inferred	DNA Fragmentation
Manganese (Mn)	Not Specified	
Mercury (Hg)	Inferred	DNA Fragmentation
Nickel (Ni) *	Not Specified	
Selenium (Se) *	Inferred	DNA Fragmentation
Uranium (U)	Not Specified	
Vanadium (V) *	Not Specified	
Zinc (Zn) *	Inferred	DNA Fragmentation

Note. Table 2 summarizes genotoxic evidence across studies investigating heavy metals in ALS pathology, all of which were conducted in confirmed ALS patients or employed ALS-specific experimental models. The “DNA Damage” column includes three categories: “Yes”, “Inferred”, and “Not Specified”. “Yes” indicates direct observation of DNA damage or specific genotoxic endpoints, which collectively define the term “DNA damage”, as described by Shoeb et al., 2023 [24]. “Inferred” indicates biomarkers or test outcomes strongly suggesting DNA damage without explicit mention. “Not Specified” indicates no observed DNA damage, genotoxic endpoints, or biomarkers suggesting its occurrence. The “Evidence” column lists specific genotoxic endpoints observed for each metal in studies conducted in confirmed ALS patients or ALS-specific experimental models. DNA fragmentation was inferred for cadmium (Cd). Metals with potential therapeutic effects for ALS are marked with ‘*’. Cell highlights: green (Yes), yellow (Inferred), red (Not Specified). A lack of shading indicates no evidence was found.

**Table 3:** Metal Exposure Toxicity Unique to ALS Mutations.

Gene	Mutation	Co	Cu	Fe	Pb	Mn	Hg	Ni	Se	Zn	Total
*C9orf72*	*G4C2*							1			1
*SOD1*	*A4V*		4						1	1	6
*SOD1*	*D76Y*									1	1
*SOD1*	*D83H*	1									1
*SOD1*	*D124V*									2	2
*SOD1*	*D125H*		1							1	2
*SOD1*	*G37R*		6	1						3	10
*SOD1*	*G41D*		1								1
*SOD1*	*G85R*	2	3							3	8
*SOD1*	*G86R*		1	1							2
*SOD1*	*G93A*	2	8	6	1		1	1		9	28
*SOD1*	*G93C*		1								1
*SOD1*	*G93R*			1							1
*SOD1*	*H46R*	2	3							3	8
*SOD1*	*H48Q*	1	1								2
*SOD1*	*H63A*	2								1	3
*SOD1*	*H63E*	2								1	3
*SOD1*	*H80C*	1									1
*SOD1*	*H80R*		1								1
*SOD1*	*I113T*		1								1
*SOD1*	*L67P*									1	1
*SOD1*	*L106V*		1							1	2
*SOD1*	*L126S*		1							1	2
*SOD1*	*S134N*									1	1
*TBK1*	*deletion in c.1852_1854delGAA: p.E618del*						1				1
*TDP43*	*A315T*		1			1				1	3
*TUBA4A*	*A383T*								1		1
*TUBA4A*	*R320C*								1		1
	Total	13	34	9	1	1	2	2	3	30	95

Note. Table 3 summarizes ALS studies reporting altered properties or neurodegenerative effects from metal exposure in models or patients with specific mutations. Each mutation’s associated gene is listed to the left of the cell. All pathogenic mutations were identified from 227 studies meeting the inclusion criteria. In ALS patients and in vitro/in vivo models with these mutations, metal exposure leads to altered metal-binding properties, CNS metal accumulation, or worsened ALS symptoms and progression. Totals for each metal and mutation are provided. Aluminum (Al), Arsenic (As), Cadmium (Cd), Chromium (Cr), Uranium (U), and Vanadium (V) are not included, as no studies reported altered properties or neurodegenerative effects from their exposure in relevant models or patients with specific mutations. Cell highlights: green (1 study), orange (2–9 studies), red (>9 studies). A lack of shading indicates no studies were found. The bottom-right yellow cell shows the total studies included (95).

**Table 4:** Clinical and Etiological Comparison of Familial and Sporadic ALS.

Category	Familial ALS (fALS)	Sporadic ALS (sALS)
Prevalence	~5–10% of cases	~90–95% of cases
Age of Onset	Average onset in 40 –50 s	Average onset in 50–60 s
Genetic Contribution	Strong; linked to SOD1, FUS, TARDBP, C9orf72 mutations	Multifactorial; less often linked to identifiable mutations
Environmental Factors	May act as secondary triggers	Often significant; includes metals and pesticides
Family History	Frequently positive; may include FTD	Rarely positive
Progression Rate	Variable: some mutations linked to faster decline	Generally slower; varies individually
Biomarkers	Detectable via genetic testing	No confirmed biomarkers; clinical diagnosis

Note. Table 4 summarizes key clinical and etiological differences between familial ALS (fALS) and sporadic ALS (sALS), including prevalence, age of onset, genetic and environmental contributions, family history, disease progression, and the availability of biomarkers.

## Data Availability

Data can be available on request.

## Supplementary Materials

The following supporting information can be downloaded at: https://www.mdpi.com/article/10.3390/toxics13060493/s1, Table S1. Search Strategy; Table S2. Study Eligibility Criteria (PECOS); Table S3. Genotoxic Endpoints Reported in ALS Patients or ALS Model Studies with Metal Exposure; Table S4. Metal Biomarkers and Test Outcomes in ALS; Table S5. ALS Mutations Associated with Metal Exposure [25,85,86,87,88,89,90,91,92,93,94,95,96,97,98,99,100,101,102,103,104,105,106,107,108,109,110,111,112,113,114,115,116,117,118,119,120,121,122,123,124,125,126,127,128,129,130,131,132].
