# Supplementary material for: Metal-Induced Genotoxic Events: Possible Distinction Between Sporadic and Familial ALS
Source: Toxics. 2025 Jun 12;13(6):493. doi: 10.3390/toxics13060493 (PMC12197494; doi:10.3390/toxics13060493)
Supplement: Supplementary file 1 [file toxics-13-00493-s001.zip › toxics-3628888 - Supplementary.pdf]

Supplementary Tables

Table S1. Search Strategy

Database	Strategy	Run Date	Records
PubMed, Scopus, Embase, Medline, Google Scholar, Environmental Science Collection	Search: [Metal Name] Amyotrophic lateral sclerosis, [Metal Name] ALS<br><br>Filters: from 1000/1/1 - 2024/5	Up to May 2024	304

Note. Supplementary Table S1 outlines the search strategy used to identify relevant literature on heavy metal exposure–induced genotoxicity in ALS patients or ALS-specific experimental models. It details the databases searched, the search terms applied, the date range, and the total number of records retrieved as of May 2024.

Table S2. Study Eligibility Criteria (PECOS)

	Inclusion Criteria	Exclusion Criteria
Population	• Human populations of any age or sex diagnosed with ALS.<br>• In vitro and in vivo models utilizing any cell line, tissue, or organism that mimics or exhibits ALS-like symptoms or pathology	• Human populations with Frontotemporal dementia (FTD), Motor Neuron Disease (MND), Gulf War illness, Alzheimer's, or Parkinson's<br>• Human populations without ALS diagnosis or symptoms<br>• In vitro and in vivo models without ALS symptoms or pathology
Exposure	• Measured or reported exposure to at least one metal of interest	• Studies did not independently analyze metal effects (ratios/mixtures).
Comparators	• Evidence of DNA damage<br>• Neurodegenerative effects	• NA

	• Altered metal concentrations or binding properties	
Outcomes	• Included significant associations ($p < 0.05$, 95% CI or higher) or author judgment if no statistical test was used.	• Excluded insignificant associations ($p > 0.05$, <95% CI) or author judgment if no statistical test was used.
Study design	• Observational studies and experimental research	• Review articles

Note. Supplementary Table S2 outlines the study eligibility criteria using the PECOS framework (Population, Exposure, Comparators, Outcomes, and Study Design) for selecting studies on heavy metal-associated ALS. It details the inclusion and exclusion criteria applied to human, in vitro, and in vivo studies, as well as the conditions for exposure, comparators, and outcomes.

Table S3. Genotoxic Endpoints Reported in ALS Patients or ALS Model Studies with Metal Exposure

Metal	Chromosomal Damage	DNA Methylation	DNA-Protein Cross-linking	Micronuclei Formation	Sister-Chromatid Exchange	DNA Repair Inhibition	Gene Mutation	Chromosomal Aberrations	DNA Fragmentation	DNA Strand Breaks	Telomere Alteration
Aluminum (Al)	Not Specified	Not Specified	Not Specified	Not Specified	Not Specified	Not Specified	Not Specified	Not Specified	Not Specified	Not Specified	Not Specified
Arsenic (As)	Not Specified	Not Specified	Not Specified	Not Specified	Not Specified	Not Specified	Not Specified	Not Specified	Not Specified	Not Specified	Not Specified
Cadmium (Cd)	Not Specified	Yes	Not Specified	Not Specified	Not Specified	Yes	Not Specified	Not Specified	Inferred	Not Specified	Not Specified
Chromium (Cr)	Not Specified	Not Specified	Not Specified	Not Specified	Not Specified	Not Specified	Not Specified	Not Specified	Not Specified	Not Specified	Not Specified
Cobalt (Co)	Not Specified	Not Specified	Not Specified	Not Specified	Not Specified	Not Specified	Not Specified	Not Specified	Inferred	Not Specified	Not Specified
Copper* (Cu)	Not Specified	Not Specified	Not Specified	Not Specified	Not Specified	Not Specified	Not Specified	Not Specified	Inferred	Not Specified	Not Specified
Iron* (Fe)	Not Specified	Not Specified	Not Specified	Not Specified	Not Specified	Not Specified	Not Specified	Not Specified	Inferred	Not Specified	Not Specified
Lead (Pb)	Not Specified	Not Specified	Not Specified	Not Specified	Not Specified	Not Specified	Not Specified	Not Specified	Not Specified	Not Specified	Not Specified
Manganese (Mn	Not Specified	Not Specified	Not Specified	Not Specified	Not Specified	Not Specified	Not Specified	Not Specified	Not Specified	Not Specified	Not Specified
Mercury (Hg)	Not Specified	Not Specified	Not Specified	Not Specified	Not Specified	Not Specified	Not Specified	Not Specified	Not Specified	Not Specified	Not Specified
Nickel (Ni)	Not Specified	Not Specified	Not Specified	Not Specified	Not Specified	Not Specified	Not Specified	Not Specified	Not Specified	Not Specified	Not Specified
Selenium* (Se)	Not Specified	Not Specified	Not Specified	Not Specified	Not Specified	Not Specified	Not Specified	Not Specified	Inferred	Not Specified	Not Specified
Uranium (U)	Not Specified	Not Specified	Not Specified	Not Specified	Not Specified	Not Specified	Not Specified	Not Specified	Not Specified	Not Specified	Not Specified
Vanadium (V)	Not Specified	Not Specified	Not Specified	Not Specified	Not Specified	Not Specified	Not Specified	Not Specified	Not Specified	Not Specified	Not Specified
Zinc* (Zn)	Not Specified	Not Specified	Not Specified	Not Specified	Not Specified	Not Specified	Not Specified	Not Specified	Inferred	Not Specified	Not Specified

Note. Supplementary Table S3 complements Table 2, summarizing evidence for the 11 genotoxic endpoints that define DNA damage, as per Shoeb et al., 2023, specifically in studies of heavy metal exposure in ALS patients or ALS-specific experimental models.

Table S4. Metal Biomarkers and Test Outcomes in ALS

Metal	Biomarkers/Test Outcome	References
**Aluminum (Al)**	No matching biomarker	N/A
**Arsenic (As)**	No matching biomarker	N/A
**Cadmium (Cd) (11)**	Increased p53 activation	Forcella et al., 2020
	Decreased DNA repair enzymes	Forcella et al., 2020
	Increased heat shock proteins (HSPs) expression	Forcella et al., 2020
	Increased metallothionein (MTs) expression	Forcella et al., 2020; Urani et al., 2018; Polykretis et al., 2019
	Up-regulation of HMOX1	Forcella et al., 2020; Urani et al., 2018
	Up-regulation of ZnT-1	Forcella et al., 2020
	Up-regulation of Ferritin	Forcella et al., 2020
	Up-regulation of S100A2	Forcella et al., 2020
	Decreased SOD1 enzyme activity	Bovio et al., 2021
	Down-regulation of NEU4	Urani et al., 2018
	Hyper/hypo methylation	Freydenzon et al., 2022
**Chromium (Cr)**	No matching biomarker	N/A
**Cobalt (Co) (7)**	Increased Hif1α expression	Xu et al., 2011
	Increased HO1 expression	Xu et al., 2011
	Increased UCP4 expression	Xu et al., 2011
	Increased histone protein release	Xu et al., 2011
	Increased caspase 3 activation	Xu et al., 2011
	Increased Aif translocation	Xu et al., 2011
	Increased cytochrome c translocation	Xu et al., 2011
**Copper (Cu)* (2)**	Increased metallothionein (MTs) expression	Tokuda et al., 2007; Tokuda et al., 2014
	Increased oxidized lipids such as HNE and HHE	Tokuda et al., 2007
**Iron (Fe)* (3)**	Increased TNF-α converting enzyme (TACE) activity and TNF-α secretion	Lee et al., 2015

	Increased iron storage proteins (TfR1, ferritin L, ferritin H, PCBP1)	Halon-Golabek et al., 2024
	Decreased proteins responsible for iron export (TAU, APP, FPN1)	Halon-Golabek et al., 2024
**Lead (Pb) (5)**	HFE Polymorphisms (C282Y, H63D)	Eum et al., 2015; Eum et al., 2013
	GSTP1 Polymorphism	Eum et al., 2015
	ALAD Polymorphisms (ALAD2, Msp1)	Kamel et al., 2003
	Increased insoluble TDP43	Ash et al., 2019
	Increases VEGF expression	Barbeito et al., 2010
**Manganese (Mn)**	No matching biomarker	N/A
**Mercury (Mg) (1)**	Increased insoluble TDP43	Ash et al., 2019
**Nickel (Ni) (1)**	Increased SOD1 accumulation	Stamenković et al., 2017
**Selenium (Se)* (4)**	Decreased levels of PARP	Maraldi et al., 2011
	Increased caspase activation	Maraldi et al., 2011
	Increased SOD1 translocation	Maraldi et al., 2011
	Activation of the DAF-16 transcription factor	Estevez et al., 2014
**Uranium (U)**	No matching biomarker	N/A
**Vanadium (V)**	No matching biomarker	N/A
**Zinc (Zn)* (9)**	Increased metallothionein (MTs) expression	Tokuda et al., 2007; Puttaparthi et al., 2002; Groeneveld et al., 2003
	Increased oxidized lipids such as HNE and HHE	Tokuda et al., 2007; Kim et al., 2009
	Decreased ZnT3 and ZnT6 protein levels	Kaneko et al., 2015
	Formation of amyloid aggregates	Baziyar et al., 2022
	Upregulation of NMDA receptors	Nutini et al., 2011
	Increased intraneuronal Ca^{2+} levels	Nutini et al., 2011
	Inhibition of BDNF	Post et al., 2008
	Depletion of endogenous TDP-43 expression	Caragounis et al., 2010
	Formation of TDP43 positive inclusions	Caragounis et al., 2010

Note. Supplementary Table S4 summarizes test outcomes and potential biomarkers based on studies of heavy metal exposure in ALS patients or ALS-specific experimental models. Metals with potential therapeutic effects for ALS are marked with '*', and the number of test outcomes is shown as (#) next to each metal's name.

Table S5. ALS Mutations Associated with Metal Exposure

Metal	ALS Mutations	References
Aluminum (Al)	N/A	N/A
Arsenic (As)	N/A	N/A
Cadmium (Cd)	N/A	N/A
Chromium (Cr)	N/A	N/A
Cobalt (Co)	**SOD1**(G85R, G93A, G37R, H46R, H48Q, H63A, H63E, H80C, D83H)	Lyons et al., 1996; Xu et al., 2011; Lyons et al., 2000
Copper (Cu)	**SOD1**(G93A, G37R, A4V, G85R, G86R, H46R, L106V, G41D, H48Q, G93C, I113T, H80R, D125H, L126S), **TDP43** (A315T)	Enge et al., 2017; Tokuda et al., 2014; Watanabe et al., 2007; Kiaei et al., 2004; Carrì et al., 1994; Corson et al., 1998; Enge et al., 2018; Kreuzer et al., 2020; Tokuda et al., 2013; Roberts et al., 2014; Soon et al., 2011; Dang et al., 2014; Bourassa et al., 2014; Hilton et al., 2016; Hilton et al., 2018; Lyons et al., 1996; Ihara et al., 2005
Iron (Fe)	**SOD1**(G93A, G86R, G37R, G93R)	Hadzhieva et al., 2013; Moreau et al., 2018; Jeong et al., 2009; Consales et al., 2019; Wang et al., 2011; Kupershmidt et al., 2009; Lee et al., 2015; Popović-Bijelić et al., 2016; Soll et al., 2021
Lead (Pb)	**SOD1**(G93A)	Barbeito et al., 2010
Manganese (Mn)	**TDP43** (A315T)	Dang et al., 2014
Mercury (Hg)	**TBK1** (deletion in c.1852_1854delGAA: p.E618del), **SOD1**(G93A)	Magnavita et al., 2020; Johnson et al., 2011
Nickel (Ni)	**C9orf72** (G4C2 repeat motif), **SOD1**(G93A)	Jhan et al., 2021; Stamenković et al., 2017
Selenium (Se)	**TUBA4A**(R320C, A383T), **SOD1**(A4V)	Maraldi et al., 2019; Amporndanai et al., 2020

Uranium (U)	N/A	N/A
Vanadium (V)	N/A	N/A
Zinc (Zn)	**SOD1**(G37R, G93A, L67P, D76Y, L106V, L126S, A4V, H63A, H63E, H46R, G85R, D124V, D125H, S134N), **TDP43** (A315T)	McAllum et al., 2015; Lelie et al., 2011; Puttaparthi et al., 2002; Lyons et al., 1996; Groeneveld et al., 2003; Kaneko et al., 2015; Baziyar et al., 2022; Nutini et al., 2011; Tiwari et al., 2005; Ermilova et al., 2005; Kim et al., 2009; Hayward et al., 2002; Enge et al., 2017; Dang et al., 2014

Note. Supplementary Table S5 complements Table 3 and details observed in ALS patients or ALS-specific experimental models with documented metal exposure, along with their respective citations.

Reference 25, 85-132 are cited in the supplementary materials

25. Figueroa-Romero, C.; Mikhail, K.A.; Gennings, C.; Curtin, P.; Bello, G.A.; Botero, T.M.; Goutman, S.A.; Feldman, E.L.; Arora, M.; Austin, C. Early life metal dysregulation in amyotrophic lateral sclerosis. Ann. Clin. Transl. Neurol. 2020, 7, 872–882.

85. Forcella, M .; Lau, P.O.U.I.; Oldani, M.; Melchioretto, P.; Bogni, A.; Gribaldo, L.; Fusi, P.; Urani, C. Neuronal specific and non-specific responses to cadmium possibly involved in neurodegeneration: A toxicogenomics study in a human neuronal cell model. Neurotoxicology 2020, 76, 162–173.

86. Urani, C .; Forcella, M.; Pierre, L.; Alessia, B.; Melchioretto, P.; Laura, G.; Fusi, P. Toxicogenomics reveals neuronal specific and non-specific responses to cadmium possibly involved in neurodegeneration. Altex. Ethik 2018, 7, 236.

87. Polykretis, P .; Cencetti, F.; Donati, C.; Luchinat, E.; Banci, L. Cadmium effects on superoxide dismutase 1 in human cells revealed by NMR. Redox Biol. 2019, 21, 101102.

88. Bovio, F .; Sciandrone, B.; Urani, C.; Fusi, P.; Forcella, M.; Regonesi, M.E. Superoxide dismutase 1 (SOD1) and cadmium: A three models approach to the comprehension of its neurotoxic effects. Neurotoxicology 2021, 84, 125–135.

89. Freydenzon, A .; Nabais, M.F.; Lin, T.; Williams, K.L.; Wallace, L.; Henders, A.K.; Blair, L.P.; Wray, N.R.; Pamphlett, R.; McRae, A.F. Association between DNA methylation variability and self-reported exposure to heavy metals. Sci. Rep. 2022, 12, 10582.

90. Xu, R.; Wu, C.; Zhang, X.; Zhang, Q.; Yang, Y.; Yi, J.; Yang, R.; Tao, Y. Linking hypoxic and oxidative insults to cell death mechanisms in models of ALS. Brain Res. 2011, 1372, 133–144.

91. Lee, J.K.; Shin, J.H.; Gwag, B.J.; Choi, E.J. Iron accumulation promotes TACE-mediated TNF-α secretion and neurodegeneration in a mouse model of ALS. Neurobiol. Dis. 2015, 80, 63–69.

92. Halon-Golabek, M.; Flis, D.J.; Zischka, H.; Akdogan, B.; Wieckowski, M.R.; Antosiewicz, J.; Ziolkowski, W. Amyotrophic lateral sclerosis associated disturbance of iron metabolism is blunted by swim training-role of AKT signaling pathway. Biochim. Biophys. Acta (BBA)-Mol. Basis Dis. 2024, 1870, 167014.

93. Eum, K.D.; Seals, R.M.; Taylor, K.M.; Grespin, M.; Umbach, D.M.; Hu, H.; Sandler, D.P.; Kamel, F.; Weisskopf, M.G. Modification of the association between lead exposure and amyotrophic lateral sclerosis by iron and oxidative stress related gene polymorphisms. Amyotroph. Lateral Scler. Front. Degener. 2015, 16, 72–79.

94. Eum, K.D.; Seals, R.; Grespin, M.; Umbach, D.; Sandler, D.; Hu, H.; Kamel, F.; Weisskopf, M.G. Interaction Between HFE Polymorphisms and Cumulative Lead Exposure on the Risk of Amyotrophic Lateral Sclerosis. ISEE Conference Abstracts 2013 Vol. 2013 Issue 1 Pages 4638. DOI: doi:10.1289/isee.2013.P-2-26-13

95. Kamel, F.; Umbach, D.M.; Lehman, T.A.; Park, L.P.; Munsat, T.L.; Shefner, J.M.; Sandler, D.P.; Hu, H.; Taylor, J.A. Amyotrophic lateral sclerosis, lead, and genetic susceptibility: Polymorphisms in the delta-aminolevulinic acid dehydratase and vitamin D receptor genes. Environ. Health Perspect. 2003, 111, 1335–1339.

96. Barbeito, A.G.; Martinez-Palma, L.; Vargas, M.R.; Pehar, M.; Mañay, N.; Beckman, J.S.; Barbeito, L.; Cassina, P. Lead exposure stimulates VEGF expression in the spinal cord and extends survival in a mouse model of ALS. Neurobiol. Dis. 2010, 37, 574–580.

97. Stamenković, S; Dučić, T; Stamenković, V; Kranz, A.; Andjus, P.R. Imaging of glial cell morphology, SOD1 distribution and elemental composition in the brainstem and hippocampus of the ALS hSOD1G93A rat. Neuroscience 2017, 357, 37–55.

98. Maraldi, T.; Riccio, M.; Zambonin, L.; Vinceti, M.; De Pol, A.; Hakim, G. Low levels of selenium compounds are selectively toxic for a human neuron cell line through ROS/RNS increase and apoptotic process activation. Neurotoxicology 2011, 32, 180–187.

99. Estevez, A.O.; Morgan, K.L.; Szewczyk, N.J.; Gems, D.; Estevez, M. The neurodegenerative effects of selenium are inhibited by FOXO and PINK1/PTEN regulation of insulin/insulin-like growth factor signaling in Caenorhabditis elegans. Neurotoxicology 2014, 41, 28–43.

100. Puttaparthi, K.; Gitomer, W.L.; Krishnan, U.; Son, M.; Rajendran, B.; Elliott, J.L. Disease progression in a transgenic model of familial amyotrophic lateral sclerosis is dependent on both neuronal and non-neuronal zinc binding pro-teins. J. Neurosci. 2002, 22, 8790–8796.

101. Groeneveld, G.J.; van Weenen, J.D.L.; Van Muiswinkel, F.L.; Veldman, H.; Veldink, J.H.; Wokke, J.H.J.; Bär, P.R.; Van Den Berg, L.H. Zinc amplifies mSOD1-mediated toxicity in a transgenic mouse model of amyotrophic lateral sclerosis. Neurosci. Lett. 2003, 352, 175–178.

102. Baziyar, P.; Seyedalipour, B.; Hosseinkhani, S. Zinc binding loop mutations of hSOD1 promote amyloid fibrils under physiological conditions: Implications for initiation of amyotrophic lateral sclerosis. Biochimie 2022, 199, 170–181.

103. Nutini, M .; Frazzini, V.; Marini, C.; Spalloni, A.; Sensi, S.L.; Longone, P. Zinc pre-treatment enhances NMDAR-mediated excitotoxicity in cultured cortical neurons from SOD1G93A mouse, a model of amyotrophic lateral sclerosis. Neuropharmacology 2011, 60, 1200–1208.

104. Isabel Post, J .; Karl Eibl, J.; Michiel Ross, G. Zinc induces motor neuron death via a selective inhibition of brain-derived neurotrophic factor activity. Amyotroph. Lateral Scler. 2008, 9, 149–155.

105. Caragounis, A .; Price, K.A.; Soon, C.P.; Filiz, G.; Masters, C.L.; Li, Q.X.; Crouch, P.J.; White, A.R. Zinc induces depletion and aggregation of endogenous TDP-43. Free Radic. Biol. Med. 2010, 48, 1152–1161.

106. Lyons, T.J.; Liu, H.; Goto, J.J.; Nersissian, A.; Roe, J.A.; Graden, J.A.; Café, C.; Ellerby, L.M.; Bredesen, D.E.; Gralla, E.B.; et al. Mutations in copper-zinc superoxide dismutase that cause amyotrophic lateral sclerosis alter the zinc binding site and the redox behavior of the protein. Proc. Natl. Acad. Sci. USA 1996, 93, 12240–12244.

107. Lyons, T.J.; Nersissian, A.; Huang, H.; Yeom, H.; Nishida, C.R.; Graden, J.A.; Gralla, E.B.; Valentine, J.S. The metal binding properties of the zinc site of yeast copper-zinc superoxide dismutase: Implications for amyotrophic lateral scle-rosis. JBIC J. Biol. Inorg. Chem. 2000, 5, 189–203.

108. Watanabe, S .; Nagano, S.; Duce, J.; Kiaei, M.; Li, Q.X.; Tucker, S.M.; Tiwari, A.; Brown, R.H.; Beal, M.F.; Hayward, L.J.; et al. Increased affinity for copper mediated by cysteine 111 in forms of mutant superoxide dismutase 1 linked to amyotrophic lateral sclerosis. Free Radic. Biol. Med. 2007, 42, 1534–1542.

109. Kiaei, M .; Bush, A.I.; Morrison, B.M.; Morrison, J.H.; Cherny, R.A.; Volitakis, I.; Beal, M.F.; Gordon, J.W. Genetically decreased spinal cord copper concentration prolongs life in a transgenic mouse model of amyotrophic lateral sclerosis. J. Neurosci. 2004, 24, 7945–7950.

110. Carrì; MT ; Battistoni, A.; Polizio, F.; Desideri, A.; Rotilio, G. Impaired copper binding by the H46R mutant of human Cu, Zn superoxide dismutase, involved in amyotrophic lateral sclerosis. FEBS Lett. 1994, 356, 314–316.

111. Corson, L.B .; Strain, J.J.; Culotta, V.C.; Cleveland, D.W. Chaperone-facilitated copper binding is a property common to several classes of familial amyotrophic lateral sclerosis-linked superoxide dismutase mutants. Proc. Natl. Acad. Sci. USA 1998, 95, 6361–6366.

112. Kreuzer, M .; Stamenković; S; Chen, S.; Andjus, P.; Dučić, T. Lipids status and copper in a single astrocyte of the rat model for amyotrophic lateral sclerosis: Correlative synchrotron-based X-ray and infrared imaging. J. Biophotonics 2020, 13, e202000069.

113. Tokuda, E .; Okawa, E.; Watanabe, S.; Ono, S.I.; Marklund, S.L. Dysregulation of intracellular copper homeostasis is common to transgenic mice expressing human mutant superoxide dismutase-1s regardless of their copper-binding abilities. Neurobiol. Dis. 2013, 54, 308–319.

114. Dang, T.N .; Lim, N.K.; Grubman, A.; Li, Q.X.; Volitakis, I.; White, A.R.; Crouch, P.J. Increased metal content in the TDP-43A315T transgenic mouse model of frontotemporal lobar degeneration and amyotrophic lateral sclerosis. Front. Aging Neurosci. 2014, 6, 15.

115. Bourassa, M.W .; Brown, H.H.; Borchelt, D.R.; Vogt, S.; Miller, L.M. Metal-deficient aggregates and diminished copper found in cells expressing SOD1 mutations that cause ALS. Front. Aging Neurosci. 2014, 6, 110.

116. Hilton, J.B .; White, A.R.; Crouch, P.J. Endogenous Cu in the central nervous system fails to satiate the elevated requirement for Cu in a mutant SOD1 mouse model of ALS. Metallomics 2016, 8, 1002–1011.

117. Hilton, J.B .; Kysenius, K.; White, A.R.; Crouch, P.J. The accumulation of enzymatically inactive cuproenzymes is a CNS-specific phenomenon of the SOD1G37R mouse model of ALS and can be restored by overexpressing the human copper transporter hCTR1. Exp. Neurol. 2018, 307, 118–128.

118. Consales, C .; Panatta, M.; Butera, A.; Filomeni, G.; Merla, C.; Carrì, M.T.; Marino, C.; Benassi, B. 50-Hz magnetic field impairs the expression of iron-related genes in the in vitro SOD1G93A model of amyotrophic lateral sclerosis. Int. J. Radiat. Biol. 2019, 95, 368–377.

119. Wang, Q .; Zhang, X.; Chen, S.; Zhang, X.; Zhang, S.; Youdium, M.; Le, W. Prevention of motor neuron degeneration by novel iron chelators in SOD1G93A transgenic mice of amyotrophic lateral sclerosis. Neurodegener. Dis. 2011, 8, 310–321.

120. Kupershmidt, L .; Weinreb, O.; Amit, T.; Mandel, S.; Carri, M.T.; Youdim, M.B. Neuroprotective and neuritogenic activities of novel multimodal iron-chelating drugs in motor-neuron-like NSC-34 cells and transgenic mouse model of amyotrophic lateral sclerosis. FASEB J. 2009, 23, 3766–3779.

121. Popović-Bijelić, A ; Mojović; M; Stamenković; S; Jovanović; M; Selaković; V; Andjus, P.; Bačić, G. Iron-sulfur cluster damage by the superoxide radical in neural tissues of the SOD1G93A ALS rat model. Free Radic. Biol. Med. 2016, 96, 313–322.

122. Soll, M .; Goldshtein, H.; Rotkopf, R.; Russek-Blum, N.; Gross, Z. A synthetic SOD/Catalase mimic compound for the treatment of ALS. Antioxidants 2021, 10, 827.

123. Magnavita, N .; Sabatelli, M.; Scoditti, E.; Chirico, F. Personalized prevention in mercury-induced amyotrophic lateral sclerosis: A case report. Appl. Sci. 2020, 10, 7839.

124. Johnson, F.O .; Yuan, Y.; Hajela, R.K.; Chitrakar, A.; Parsell, D.M.; Atchison, W.D. Exposure to an environmental neurotoxicant hastens the onset of amyotrophic lateral sclerosis-like phenotype in human Cu2+/Zn2+ superoxide dismutase 1 G93A mice: Glutamate-mediated excitotoxicity. J. Pharmacol. Exp. Ther. 2011, 338, 518–527.

125. Jhan, C.R .; Satange, R.; Wang, S.C.; Zeng, J.Y.; Horng, Y.C.; Jin, P.; Neidle, S.; Hou, M.H. Targeting the ALS/FTD-associated A-DNA kink with anthracene-based metal complex causes DNA backbone straightening and groove contraction. Nucleic Acids Res. 2021, 49, 9526–9538.

126. Maraldi, T .; Beretti, F.; Anselmi, L.; Franchin, C.; Arrigoni, G.; Braglia, L.; Mandrioli, J.; Vinceti, M.; Marmiroli, S. Influence of selenium on the emergence of neuro tubule defects in a neuron-like cell line and its implications for amyotrophic lateral sclerosis. Neurotoxicology 2019, 75, 209–220.

127. Amporndanai, K .; Rogers, M.; Watanabe, S.; Yamanaka, K.; O'Neill, P.M.; Hasnain, S.S. Novel Selenium-based compounds with therapeutic potential for SOD1-linked amyotrophic lateral sclerosis. eBioMedicine 2020, 59, 102980.

128. McAllum, E.J.; Roberts, B.R.; Hickey, J.L.; Dang, T.N.; Grubman, A.; Donnelly, P.S.; Liddell, J.R.; White, A.R.; Crouch, P.J. ZnII (atsm) is protective in amyotrophic lateral sclerosis model mice via a copper delivery mechanism. Neurobiol. Dis. 2015, 81, 20–24.

129. Lelie, H.L .; Liba, A.; Bourassa, M.W.; Chattopadhyay, M.; Chan, P.K.; Gralla, E.B.; Miller, L.M.; Borchelt, D.R.; Valentine, J.S.; Whitelegge, J.P. Copper and zinc metallation status of copper-zinc superoxide dismutase from amyotrophic lateral sclerosis transgenic mice. J. Biol. Chem. 2011, 286, 2795–2806.

130. Tiwari, A .; Xu, Z.; Hayward, L.J. Aberrantly Increased Hydrophobicity Shared by Mutants of Cu, Zn-Superoxide Dismutase in Familial Amyotrophic Lateral Sclerosis. J. Biol. Chem. 2005, 280, 29771–29779.

131. Ermilova, I.P .; Ermilov, V.B.; Levy, M.; Ho, E.; Pereira, C.; Beckman, J.S. Protection by dietary zinc in ALS mutant G93A SOD transgenic mice. Neurosci. Lett. 2005, 379, 42–46.

132. Hayward, L.J .; Rodriguez, J.A.; Kim, J.W.; Tiwari, A.; Goto, J.J.; Cabelli, D.E.; Valentine, J.S.; Brown, R.H. Decreased metallation and activity in subsets of mutant superoxide dismutases associated with familial amyotrophic lateral sclerosis* 210. J. Biol. Chem. 2002, 277, 15923–15931.
